# The effect of intraoperative dexmedetomidine on cognitive dysfunction after surgery: a updated meta-analysis

**DOI:** 10.1186/s13019-021-01736-z

**Published:** 2021-12-14

**Authors:** Jianli Li, Qifan Yin, Xuejiao Xun, Jinhua He, Dongdong Yu, Zhibin Wang, Junfang Rong

**Affiliations:** 1 Department of Anesthesiology, Hebei General Hospital, Shijiazhuang, 050051 Hebei Province China; 2 Department of Thoracic Surgery, Hebei General Hospital, Shijiazhuang, 050051 Hebei Province China; 3 Department of Pharmacy, Hebei General Hospital, Shijiazhuang, 050051 Hebei Province China; 4 Department of Anesthesiology, The Fifth People’s Hospital of Hengshui, Hengshui, 053000 Hebei Province China

**Keywords:** Postoperative cognitive dysfunction, Dexmedetomidine, MMSE score, Meta-analysis, Perioperative period

## Abstract

**Background:**

Postoperative cognitive dysfunction (POCD) is one of the most common. Neuroprotective effects of dexmedetomidine (DEX) are reported in previous studies but evidence regarding the POCD is still unclear. In order to gain latest evidence, the present study analyzes the outcomes of randomized controlled trials (RCTs) which utilized DEX with general anaesthesia perioperatively.

**Method:**

Four online databases (PubMed, Embase, the Cochrane Library, and CNKI) were used to find relevant RCTs to conduct systematic analysis. All studies comparing the incidence of POCD or MMSE score between the DEX group and the placebo or comparator group in patients undergoing general anaesthetic surgery were eligible for inclusion. Based on the inclusion and exclusion criteria, the studies were selected. This meta-analysis was performed using odds ratios (ORs) with 95% confidence intervals (CIs) for dichotomous data and standardized mean difference (SMD) and 95% CIs for continuous data as effective measures.

**Results:**

In total of 21 studies were included in this meta-analysis. The results showed that the incidence of POCD in DEX group was significantly lower than the control group on the first (OR = 0.36, 95% CI 0.24–0.54),third (OR = 0.45,95% CI 0.33–0.61) and seventh (OR = 0.40,95% CI 0.26–0.60) postoperative days; the MMSE scores in DEX group were higher than the control group on the first (SMD = 1.24, 95% CI 1.08–1.41), third(SMD = 1.09, 95%CI 0.94–1.24) and seventh (SMD = 3.28, 95% CI 1.51–5.04) postoperative days.

**Conclusions:**

Intraoperative DEX use can ameliorate the POCD of patients who received surgical operations under general anesthesia, and effectively reduce the incidence of POCD and improve MMSE score.

## Introduction

Postoperative cognitive dysfunction (POCD) is one of the most common complications affecting the central nervous system after general anaesthesia and surgery, especially in elderly patients, which is characterized by short-term cognitive decline and includes memory, mood, confusion, and sleep disorders [1]. Its clinical manifestations include cognitive dysfunction, personality change, and memory loss, mental disorders, and social impairment [2]. Because of the long anesthesia duration and the severe surgical stress, the risk of POCD in elderly patients is incredibly increased [3]. The prevalence of POCD is ranging from 10 to 60% and varies with clinical, demographic, and surgical variables, as well as the interval between surgery and assessment in older patients [4, 5]. POCD often leads to prolonged hospitalization, increased medical costs, lower likelihood of return to independent living, and increased mortality [6–8]. The elderly are vulnerable to cognitive dysfunction following operation [9–11]. The Mini Mental State Examination (MMSE) is commonly used to assess cognitive function postoperatively [12]. However, the exact mechanism of developing POCD remains unknown [13]. The majority of researchers consider that neuroinflammation exerts an crucial role in the development of POCD [14–16].

Dexmedetomidine (DEX) is a effective and highly selective α2-adrenergic receptor agonist and acts as a multifunctional drug in the treatment of various human diseases [17, 18]. A previous study has suggested that DEX is efficient in the treatment of nerve diseases through the beneficial effects of decreasing central nervous system sympathetic outflow and providing sedation and analgesia [19]. DEX treatment may improve behavioral disturbances, including aggression, agitation and cognitive dysfunction [20]. In addition, some clinical studies have indicated that DEX has analgesic, anxiolytic and anti-delirium effects without respiratory depression [17, 21]. These properties make it anappropriate option for sedation in the intensive care unit and in perioperative period.

Several basic researches with animal models have reported that DEX provide neuroprotective effects and improve cognitive function following surgery [22, 23]. In clinical studies, many have indicated beneficial effects of DEX in improving postoperative cognitive impairment while others could not observe the similar results. Therefore, it is still unclear that whether the administration of intraoperative DEX can ameliorate POCD. In order to gain latest evidence, the present study systematically and comprehensively analyzes the outcomes of RCTs which examined neurocognitive performance and MMSE scores to investigate the effects of DEX on POCD in patients after general anaesthesia.

## Materials and methods

### Search strategy

Two review authors (Jianli Liand Qifan Yin) independently searched PubMed, Embase, the Cochrane Library, China Academic Journals full-text database (CNKI) to find relevant RCTs without any language restrictions. All studies comparing the incidence of POCD or MMSE score between the DEX group and the placebo or comparator group in patients undergoing general anaesthetic surgery were eligible for inclusion. The search was updated to Dec 31, 2020. The main search terms included: (“dexmedetomidine” OR “Dex”) AND (“postoperative cognitive dysfunction” OR “POCD” OR “postoperative cognitive impairment”). The reference list was also checked for relevant articles.

### Inclusion and exclusion criteria

Studies were included according to the following criteria: (1) all of the enrolled studies were randomized controlled trials (RCTs); (2) adult patients undergoing surgery under general anaesthesia; (3) the experimental group received a single or continuously-administered intravenous dose of intraoperative DEX; the control groups received an intravenous injection of placebo or comparator; (4) the main outcomes in enrolled studies were the incidence of POCD, postoperative MMSE score; (5) all of included studies contained original data sufficient for meta-analysis.

The exclusion criteria were: (1) abstracts, letters, case reports, reviews, duplicates or nonclinical studies; (2) studied patients who underwent local or spinal-epidural anesthesia; (3) patients administered DEX compared with other sedative agents (benzodiazepines, midazolam, and propofol) were excluded; (4) articles with poor quality, Jadad score ≤ 3 points, were excluded; (5) studies with incomplete information or data, and articles for which we could not obtain the full text.

### Data extraction and quality assessment

Two researchers (Xuejiao Xun and Jinhua He) independently screened the literature, extracted data and evaluated the methodologic quality of the studies identified. If disagreement occurred, a consensus was reached after discussion with a third author. All the corresponding data including the name of first author, publication year, country, age, number of participants, type of surgery, administrations for patients, incidences of POCD, MMSE score were extracted from each selected study. We evaluated methodologic quality of the RCTs enrolled using a Jadad scale. Evaluation included randomization, allocation concealment, and blinding of implementers and participants. Studies awarded with greater than three scores were considered to be of acceptable quality. The information and data of enrolled studies were entered in a standard data extraction form. Table 1 shows the extracted contents and Jadad scores.

**Table 1:** The basic characteristics of the enrolled studies

Author	Country	Age	Number	Surgical type	Administration	Events of POCD			MMSE score (mean ± SD)			Jadad score
		Cases/controls	Cases/controls		Cases/controls	1 day after surgery	3 days after surgery	7 days after surgery	1 day after surgery	3 days after surgery	7 days after surgery	
Peng2012	China	70.6/67.2	40/40	Prostate resection	DEX/Saline	NA	5/11	NA	NA	24.3 ± 0.6/20.5 ± 0.7	NA	4
Chen2013	China	66.2/67.9	59/63	Laparoscopic cholecystectomy	DEX/Saline	9/27	NA	NA	NA	NA	27.6 ± 1.2/25.7 ± 1.5	5
Zhang2014	China	71.6/71.5	60/20	Laparoscopic surgery	DEX/Saline	NA	8/7	6/7	NA	NA	NA	4
Mohamed2014	Africa	63.9/67.8	25/25	Abdominal surgery	DEX/Saline	4/20	NA	NA	NA	NA	NA	5
Ding2015	China	NA	20/20	Laparoscopic prostatectomy	DEX/Saline	9/12	NA	NA	NA	NA	NA	5
Li2015	China	69/70	50/50	Laparoscopic cholecystectomy	DEX/Saline	10/21	NA	NA	27.9 ± 1.7/26.7 ± 1.9	NA	NA	4
Guan2015	China	65.9/68.2	30/30	Laparoscopic surgery	DEX/Saline	9/8	10/9	NA	23.35 ± 5.16/22.46 ± 4.35	27.84 ± 4.67/25.79 ± 4.90	NA	4
**Liu2015**^**a**^	**China**	**72.84/75.25**	**39/40**	**Joint replacement**	DEX/Saline	5/6	3/6	2/13	NA	NA	NA	5
**Liu2015**^**b**^	**China**	**71.23/72.81**	**60/58**	**Joint replacement**	DEX/Saline	2/9	3/6	0/3	NA	NA	NA	5
Xu2016	China	51/48.8	40/40	Orthotopic liver transplatation	DEX/Saline	8/18	NA	NA	25.87 ± 1.93/22.41 ± 2.95	NA	NA	5
Deiner2017	USA	74/74	189/201	Elective non-cardiac surgery	DEX/Saline	23/23	NA	NA	NA	NA	NA	5
Ma2017	China	49.3/48.1	25/25	Thyroid surgery	DEX/Saline	NA	NA	NA	24.86 ± 2.48/22.37 ± 2.36	25.92 ± 2.4/23.43 ± 2.38	NA	4
Xu2017	China	71.9/72.1	48/48	Ovarian cystectomy	DEX/Saline	0/2	1/2	0/0	NA	NA	NA	5
Kong2018	China	67.8/68.9	60/60	Cancer surgery	DEX/Saline	6/17	NA	NA	27.1 ± 0.69/23.14 ± 0.71	28.53 ± 0.62/24.8 ± 0.55	NA	4
Zheng2018	China	42.3/42.4	40/40	Coronary artery bypass grafting	DEX/Saline	1/10	NA	NA	20.6 ± 0.5/17.5 ± 0.9	25.1 ± 0.7/21.3 ± 1.1	28.6 ± 0.3/23.8 ± 0.7	4
Zhang2019	China	73.76/74.09	80/60	Radical colorectal cancer surgery	DEX/Saline	7/13	0/8	NA	26.76 ± 1.67/24.15 ± 1.98	28.11 ± 2.01/26.09 ± 1.78	NA	4
Cheng2019	China	71/70	269/266	Gastro-intestinal laparotomy	DEX/Saline	NA	40/65	31/49	NA	NA	NA	5
Shi Haixia2020	China	66.4/67.6	40/40	Thoracoscopic lobectomy	DEX/Saline	NA	4/12	NA	NA	27.6 ± 2.3/24.1 ± 4.01	NA	5
Shi Hai-Xia2020	China	68.71/68.7	53/53	Thoracoscopic lobectomy	DEX/Saline	7/19	NA	NA	26.3 ± 1.27/26.09 ± 1.01	NA	NA	4
Bin2020	China	72/73	183/183	Knee hip arthroplasty	DEX/Propofol	NA	NA	NA	NA	24.3 ± 3.9/22.1 ± 4.3	25.6 ± 4.8/23.3 ± 3.3	5
Wang2020	China	68.37/68.26	60/50	Radical gastrectomy	DEX/Saline	12/20	NA	NA	27.34 ± 2.45/24.21 ± 3.11	NA	NA	4
Yan2020	China	69.5/70.4	30/30	Coronary artery bypass grafting	DEX/Saline	NA	3/9	0/5	28.4 ± 1.2/28.2 ± 1.1	24.5 ± 1.1/20.3 ± 1.3	28.2 ± 1.3/24.2 ± 1.2	4

*DEX* dexmedetomidine, *NA* not available

Liu2015^a^ and Liu2015^b^ are from same study

### Statistical analysis

We used Review Manager 5.3 to conduct the meta-analysis. For dichotomous data, odds ratios (ORs) with 95% confidence intervals (CIs) were used to express effect-size, while standardized mean difference (SMD) and 95% CIs were used for continuous data. First, we conducted a heterogeneity test on included studies using the I^2^ test. A fixed effects model was performed to conduct the meta-analysis if no heterogeneity (*P* > 0.1 or I^2^ < 50.0%) was found among the studies. If significant heterogeneity (*P* < 0.1 or I^2^ ≥ 50.0%) was found, a random effects model was applied for the meta-analysis. Bias or publication bias was evaluated as quality using funnel plots, Egger’s and Begg’s tests. A value of *P* < 0.05 was considered statistically significant.

## Results

### Study selection

**Fig. 1:**
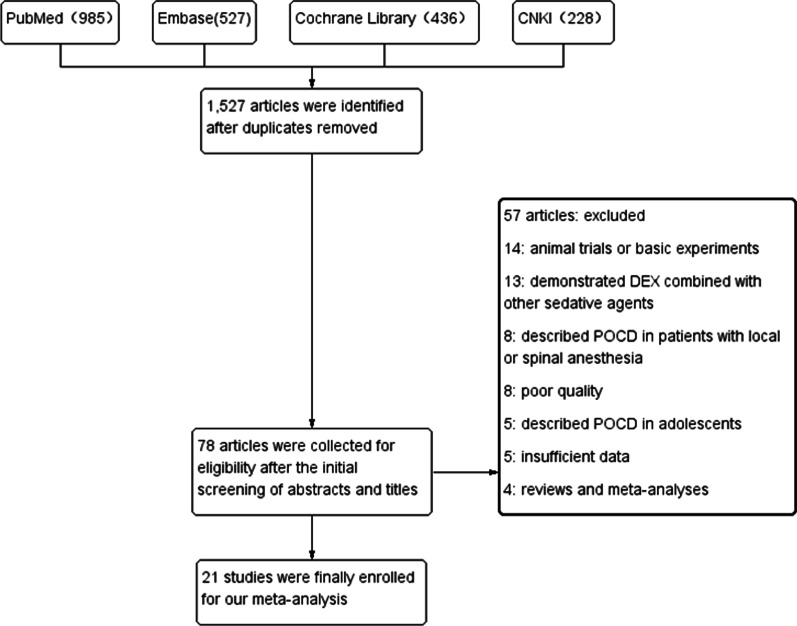
Flow diagram of the literature selection

In the initial search, a total of 1,527 potential articles were identified. After the initial screening of abstracts and titles, 1,448 articles were excluded based on the inclusion and exclusion criteria. The remaining 78 articles were collected for full text review, after the secondary screening and carefully inspection of these articles, 21 studies including 2,902 patients published between 2012 and 2020 were eventually selected for final analysis. Another 57 articles were excluded; 14 studies reported animal trials or basic experiments, 13 studies demonstrated DEX combined with other sedative agents (benzodiazepines, midazolam, and propofol), 8 studies described POCD in patients with local or spinal anesthesia, the quality of 8 studies with Jadad score ≤ 3 points were poor, 5 articles described POCD in adolescents, 5 studies did not provide complete data, 4 studies were reviews and meta-analyses. The detail processes of study screening and selection were shown in the flow diagram (Fig. 1).

### Study characteristics

All of the studies enrolled for our meta-analysis were RCTs. In total of 21 studies involved 2902 patients administered DEX (1350) and saline/comparator (1552). All included studies published between 2012 and 2020. Among them, 19 studies were from China, one study was performed in The United States and Africa, respectively. With regard to the incidence of POCD in our meta-analysis, 14 studies with 1,639 patients demonstrated the relationship between DEX and the incidence of POCD on the first day after surgery. 9 articles with 1,328 patients illustrated the association between DEX and the incidence of POCD on the third day after surgery. 5 literatures with 968 patients shown the relation between DEX and the incidence of POCD on the seventh day after surgery. Concerning the relationship between DEX and MMSE score, 9 studies with 800 patients described the relationship between DEX and the MMSE score on the first day after surgery. 8 articles with 976 patients reported the association between DEX and the MMSE score on the third day after surgery. 4 researches with 628 patients reported the association between DEX and the MMSE score on the seventh day after surgery. Dosage of DEX was in the range of 0.5 to 1.5 µg/kg followed by continuous infusion at a rate of 0.15 to 0.80 µg/kg/h. The majority of included studies investigated the elderly. The basic characteristics of each of the enrolled studies, including publication year, country, average age, number of cases, surgical type, administrations for patients, incidence of POCD, MMSE and Jadad score, were shown in Table 1. Quality of the included studies was generally moderate to good.

### The association between DEX and the incidence of POCD

**Fig. 2:**
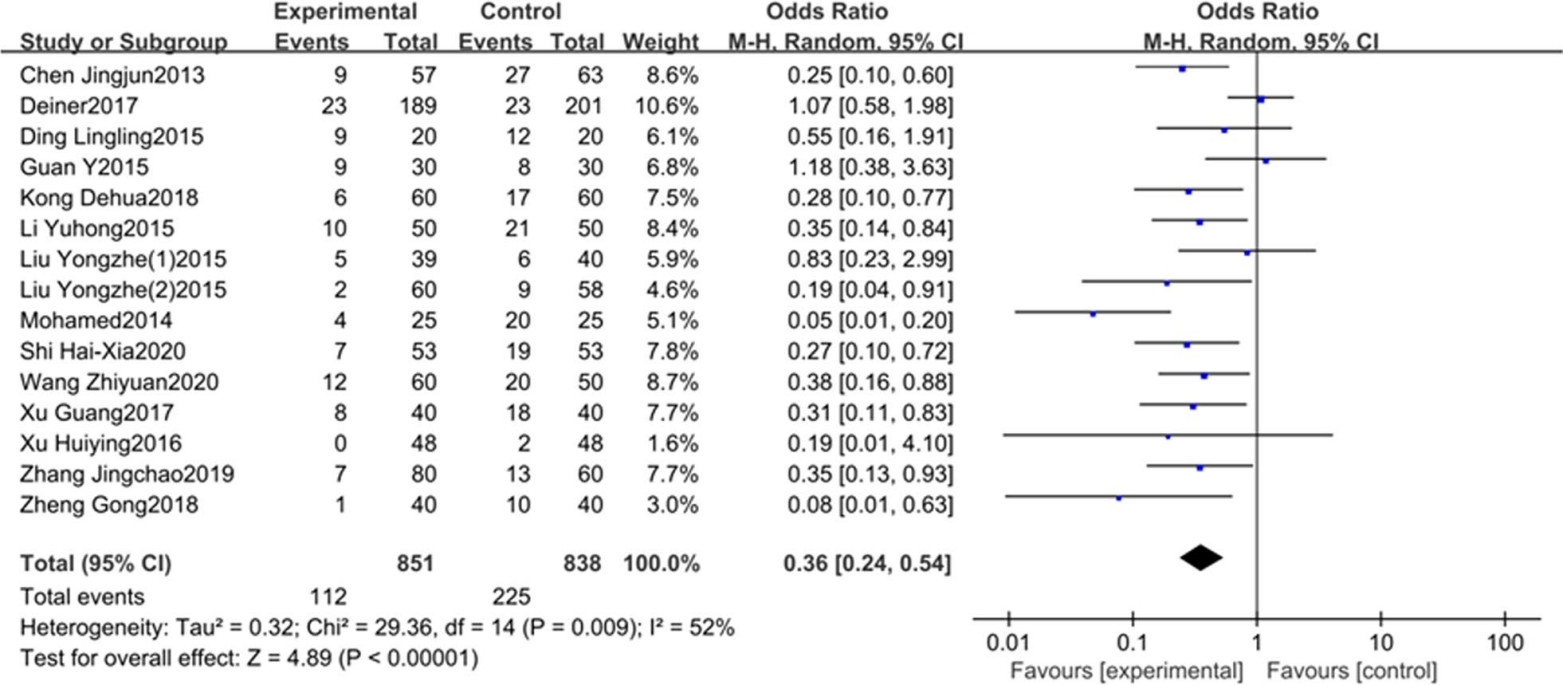
The association between DEX and the incidence of POCD on the first day after surgery

14 studies including 1639 patients reported the relationship between DEX and the incidence of POCD on the first day after surgery. There was an obvious heterogeneity between the studies (*P* = 0.009, I^2^ = 52%, Fig. 2), therefore, a random effect model was applied. Our results clearly showed that the incidence of POCD in DEX group was significantly lower than the control group (OR = 0.36, 95% CI 0.24–0.54, Fig. 2). Meta-analysis of these 14 studies depicted that the administration of intraoperative DEX could reduce the incidence of POCD on the first day after surgery compared to placebo.

**Fig. 3:**
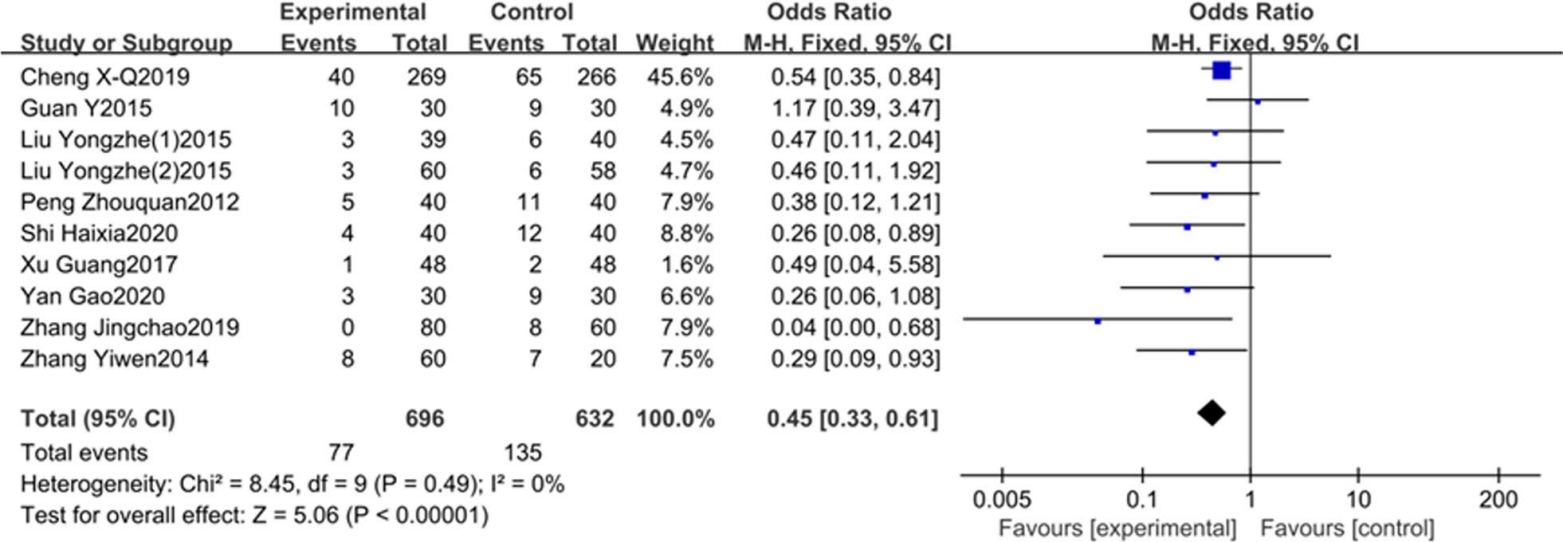
The association between DEX and the incidence of POCD on the third day after surgery

9 studies including 1328 patients elaborated the association between DEX and the incidence of POCD on the third postoperative day. Considering no obvious heterogeneity between the studies (I^2^ = 0%, *P* = 0.49, Fig. 3), a fixed-effects model was adopted. The results of our meta-analysis suggested that the incidence of POCD in DEX group was significantly lower than controls (OR = 0.45, 95% CI 0.33–0.61, Fig. 3). Meta-analysis of these 9 studies indicated that the intraoperative DEX could obviously lower the incidence of POCD on the third day after surgery compared to control group.

**Fig. 4:**
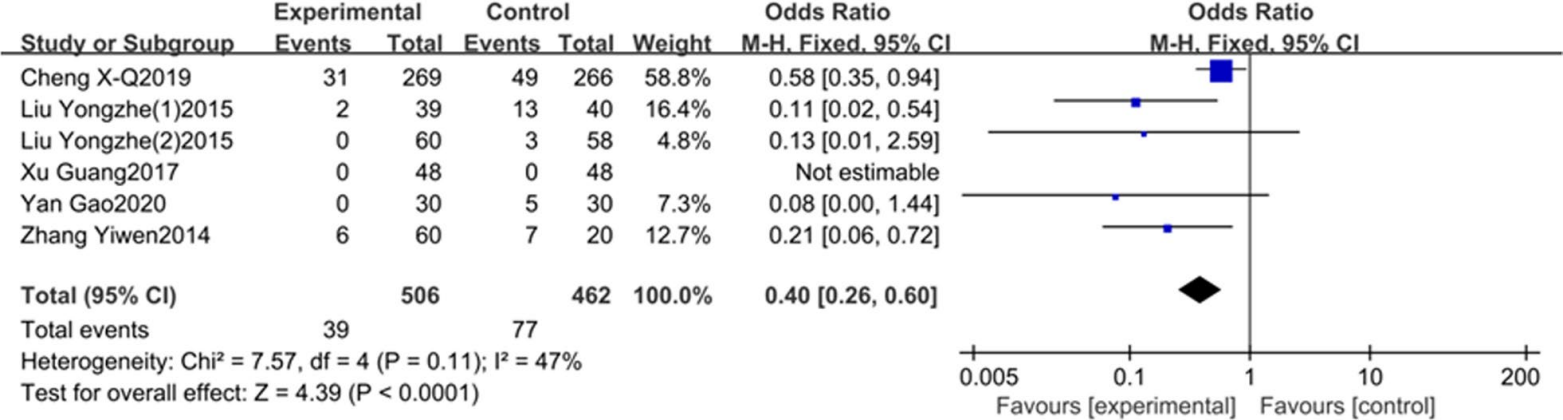
The association between DEX and the incidence of POCD on the seventh day after surgery

5literatures including 968 patients reported the incidence of POCD on the seventh day after surgery. On account of the little heterogeneity (I^2^ = 47%, *P* = 0.11, Fig. 4), we applied a fixed-effects model in this study. Our result clearly shown that the incidence of POCD in DEX group was obviously lower than the saline/comparator group on the seventh postoperative day (OR = 0.40, 95% CI 0.26–0.60, Fig. 4). Meta-analysis of these 5 studies shown that the intraoperative DEX could reduce the incidence of POCD on the seventh day after surgery compared to saline.

### The association between DEX and the postoperative MMSE score

**Fig. 5:**
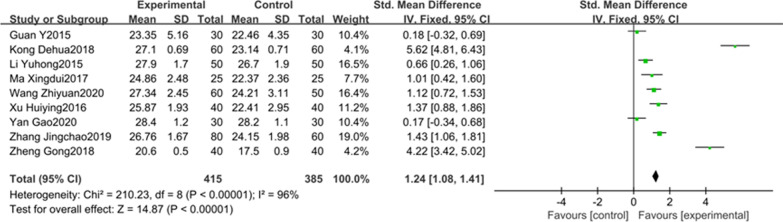
The association between DEX and the MMSE score on the first day after surgery

9 studies with 800 participants reported the relationship between DEX and the postoperative MMSE score on the first day after surgery. As we all know, MMSE is commonly used to assess cognitive function, a higher MMSE score indicates better cognitive function for postoperative patients. There was an obvious heterogeneity between the studies (*P* < 0.00001, I^2^ = 96%, Fig. 5), therefore, a random effect model was applied. Our results clearly showed that the MMSE score in DEX group was higher than the control group (SMD = 1.24, 95% CI 1.08–1.41, Fig. 5). Meta-analysis of these 9 studies revealed that the intraoperative use of DEX could improve postoperative cognitive function on the first day after surgery compared to placebo.

**Fig. 6:**
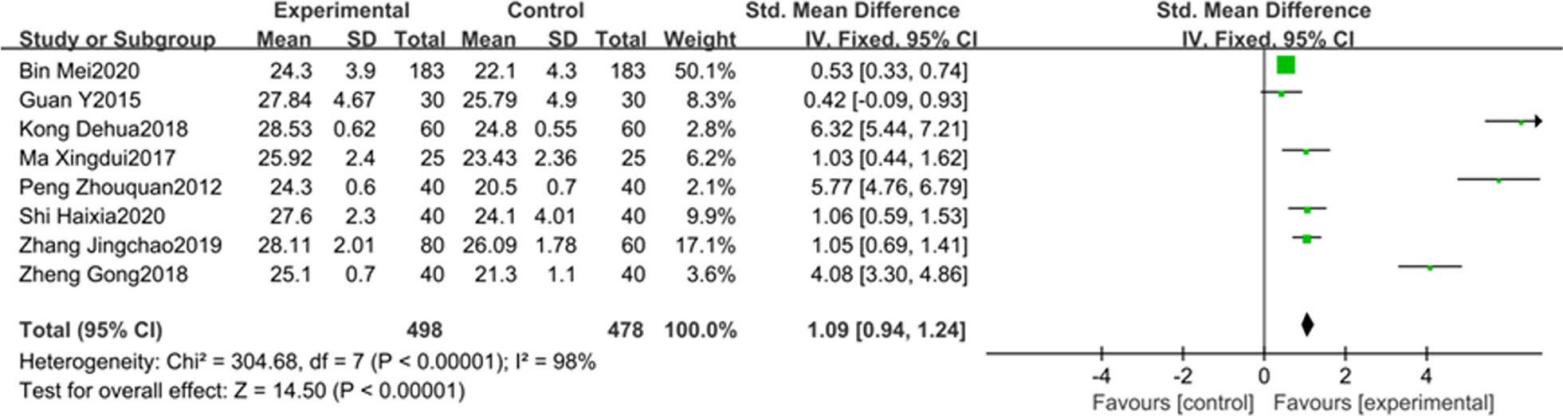
The association between DEX and the MMSE score on the third day after surgery

8studies involving 976 patients reported the MMSE score on the third postoperative day. A random effect model was adopted for meta-analysis considering the apparent heterogeneity (*P* < 0.00001, I^2^ = 98%, Fig. 6). The results from meta-analysis suggested that MMSE score was higher on the third postoperative day in the DEX group than the control group (SMD = 1.09, 95% CI 0.94–1.24, Fig. 6). Meta-analysis of these 8 studies clearly showed that the intraoperative use of DEX could improve postoperative cognitive function on the third postoperative day compared to saline.

**Fig. 7:**
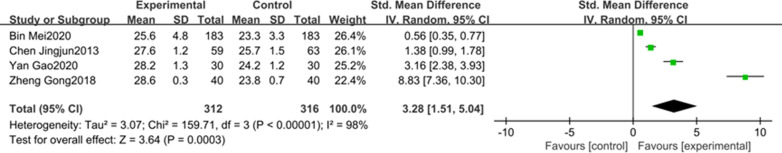
The association between DEX and the MMSE score on the seventh day after surgery

4 articles including 628 patients reported the association between DEX and the MMSE score on the seventh day after surgery. Due to a large heterogeneity (*P* < 0.00001, I^2^ = 98%, Fig. 7) between the studies, So, a random effect model was applied. Our results clearly showed that the MMSE score in DEX group was higher than the control group (SMD = 3.28, 95% CI 1.51–5.04, Fig. 7). Meta-analysis of these 4 studies indicated that the intraoperative DEX treatment was associated with better postoperative cognitive performance in comparison with saline treated controls on the seventh day after surgery.

### Publication bias

**Fig. 8:**
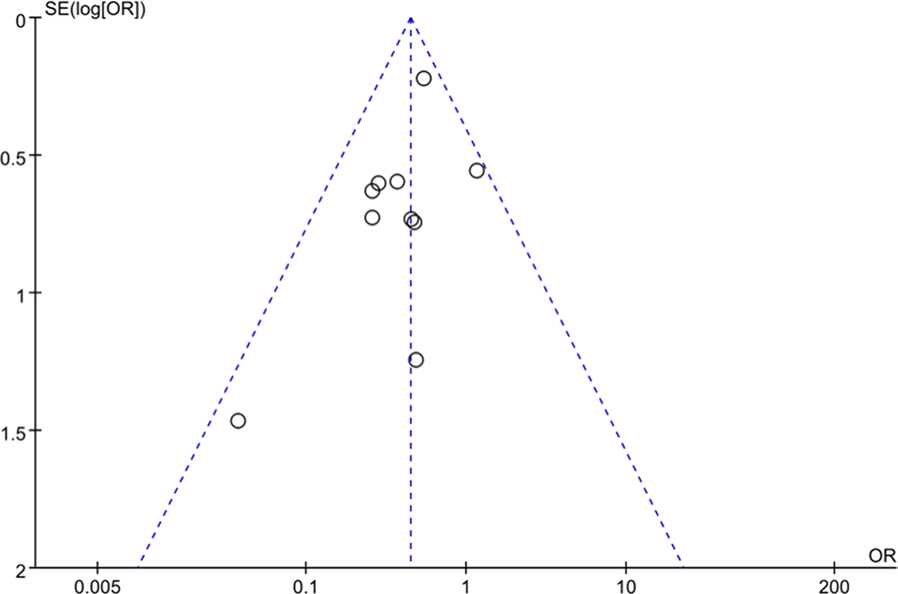
The funnel plot

A funnel plot of the 9 studies reporting the incidence of POCD on the third postoperative day was symmetrical, which indicated no publication bias (Fig. 8), with all the studies within the 95% CI of the funnel.

## Discussion

At the present study, our meta-analysis clearly showed that the intraoperative use of DEX could obviously alleviate cognitive dysfunction, lower the incidence of POCD, and improve MMSE score compared to saline treated controls on the first, third, and seventh day following surgery. We found that DEX did reduce the incidence of POCD over saline placebo. Our results similar to previous reports in which sedation with intraoperative DEX was associated with a lower incidence of POCD compared with placebo.

As the development of society and the trend of aging population aggravates, the increasing number of elderly patients are undergoing surgery, the elderly are more vulnerable to POCD. The incidence of POCD in the elderly is high, appealing to increasing interest in identifying strategies to prevent POCD. However, given its consequences, the concrete cause of POCD is still uncertain [24, 25]. It may be related to anesthesia methods, types of surgery, complications and potential cognitive differences [26]. Some previous researches have reported the main clinical manifestations of POCD includes insanity, anxiety, personality changes and memory loss [2]. Mild POCD may prolong hospitalization and increase the costs of care along with changes in memory, intelligence, verbal ability, personality, and sociability which may lead to patients’ inability to engage with work and life activities [4]. Severe POCD may cause patients to lose the ability to speak, induce personality change and Alzheimer’s disease, and impair their self-care ability [27].

Although POCD is common in older patients, the concrete pathophysiologic mechanism is still poorly understood. Our meta-analysis clearly reported that DEX can significantly reduce the incidence of POCD in patients and improve postoperative MMSE score. DEX, an imidazole derivative and a dextrorotatory isomer of medetomidine, is a novel adrenal α2 receptor agonist with high selectivity. DEX acts at the locus coeruleus in the brain stem, which contains the highest concentration ofα2adrenoceptors. It can activate the G protein on the α2 adrenergic receptor in the brain and spinal cord, and inhibit the excretion of norepinephrine and neuronal discharge, which affects the sympathetic nervous system and exerts its sedative, anti-anxiety and analgesic effects [28, 29]. There are many reasons for cognitive dysfunction in patients after surgery. Age, preoperative underlying disease, type of surgery, duration of anesthesia, postoperative infection, respiratory complications, intraoperative anesthesia, and sedation of the patients may be associated with POCD [30]. An association between POCD and the inflammatory response has also been reported. Studies examined whether systemic inflammation in response to surgical trauma leads to subsequent memory impairment and hippocampal inflammation in a mouse model of orthopaedic surgery, and found that inflammation played a critical role in the pathogenesis of POCD and could be reversed by nonspecific inhibitor of inflammation [31, 32]. The trauma of surgery stimulates the immune cascade and the release of inflammatory mediators, which may then provoke POCD. DEX can activate the cholinergic anti-inflammatory pathway and downregulate inflammatory factors to exert anti-inflammatory effects [33]. DEX exhibits anti-anxiety effects, reduces the inflammatory stress response, stabilizes hemodynamics, analgesia and reduces the incidence of adverse reactions as well as the use of sedative anesthetics [34, 35]. DEX has been indicated to inhibit inflammation and improve the characteristics of brain metabolism in organ protection studies [34, 36]. The study has found that the levels of IL-1β, IL-6 and CRP were attenuated by the administration of DEX to elderly patients undergoing general anesthesia surgery. The concentrations of IL-1β, IL-6 and CRP were significantly lower in the DEX group than in the control group following surgery. These findings indicated that DEX administration during surgery can reduce the inflammatory reaction associated with surgery and anesthetics peri-operatively [15]. The present study indicated that DEX was able to reduce the incidence of POCD, which confirmed its ability to prevent cognitive dysfunction following surgery. Moreover, a number of animal experiments have shown that DEX can lead to inflammation reduction, which may also explain how it reduces the incidence of POCD [37, 38]. Therefore, there is a certain protective effect on the brain and myocardium of patients for intraoperative DEX treatment, which significantly improves the occurrence of POCD [39].

Overall, the previous meta-analyses published by Yang et al. and Lei et al. have also found that intraoperative DEX use was associated with reduction in the incidence of POCD and increased the MMSE score compared to saline group [14, 40]. However, the included articles of their studies were too few and the quality was relatively poor. This field about POCD has seen rapid advancement during the past years, and accumulating studies have emerged. It’s necessary for us to perform a systematic review and meta-analysis to evaluate whether the results of recently published or updated trials have changed the previous analysis. In our meta-analysis, Intraoperative DEX use indeed obviously reduced the incidence of POCD, and improved MMSE score compared to saline group on the first, third, and seventh postoperative day.

Some limitations were associated with our study. First, the number of studies and the corresponding sample size were relatively limited, and the doses and methods of administration of DEX given to patients varied substantially. Secondly, the enrolled studies’ inclusion and exclusion criteria, body weight, anesthetic doses, duration of surgery, surgical blood loss and consequently the characteristics of the patient cohorts, were also varied, which might have led to heterogeneity. Clinical heterogeneity of the participants can also influence the results of a meta-analysis. Finally, we have to admit that the result of POCD was lack of strength due to the only MMSE test. Different studies had different definition of POCD, which might lead to high heterogeneity in current study.

## Conclusion

In conclusion, DEX can ameliorate the POCD of patients who received surgical operations under general anesthesia, and effectively reduce the incidence of POCD and improve MMSE score. Thus, it can serve as a kind of adjuvant drug for general anesthesia in clinical practice.

## Data Availability

The datasets used and analyzed during the current study are available from the corresponding author on reasonable request.

## Abbreviations

POCD: Postoperative cognitive dysfunction
DEX: Dexmedetomidine
RCTs: Randomized controlled trials
MMSE: Mini Mental State Examination
ORs: Odds ratios
CIs: Confidence intervals
SMD: Standardized mean difference
NA: Not available
